# Red maca (*Lepidium meyenii*) reduced prostate size in rats

**DOI:** 10.1186/1477-7827-3-5

**Published:** 2005-01-20

**Authors:** Gustavo F Gonzales, Sara Miranda, Jessica Nieto, Gilma Fernández, Sandra Yucra, Julio Rubio, Pedro Yi, Manuel Gasco

**Affiliations:** 1 Department of Biological and Physiological Sciences. Faculty of Sciences and Philosophy, Universidad Peruana Cayetano Heredia, Lima, Peru; 2 Departament of Chemistry. Faculty of Sciences and Philosophy, Universidad Peruana Cayetano Heredia, Lima, Peru; 3 Faculty of Veterinary Medicine and Animal Sciences, Universidad Peruana Cayetano Heredia, Lima, Peru; 4 Instituto de Investigaciones de la Altura. Universidad Peruana Cayetano Heredia, Lima, Peru

## Abstract

**Background:**

Epidemiological studies have found that consumption of cruciferous vegetables is associated with a reduced risk of prostate cancer. This effect seems to be due to aromatic glucosinolate content. Glucosinolates are known for have both antiproliferative and proapoptotic actions.

Maca is a cruciferous cultivated in the highlands of Peru. The absolute content of glucosinolates in Maca hypocotyls is relatively higher than that reported in other cruciferous crops. Therefore, Maca may have proapoptotic and anti-proliferative effects in the prostate.

**Methods:**

Male rats treated with or without aqueous extracts of three ecotypes of Maca (Yellow, Black and Red) were analyzed to determine the effect on ventral prostate weight, epithelial height and duct luminal area. Effects on serum testosterone (T) and estradiol (E2) levels were also assessed. Besides, the effect of Red Maca on prostate was analyzed in rats treated with testosterone enanthate (TE).

**Results:**

Red Maca but neither Yellow nor Black Maca reduced significantly ventral prostate size in rats. Serum T or E2 levels were not affected by any of the ecotypes of Maca assessed. Red Maca also prevented the prostate weight increase induced by TE treatment. Red Maca administered for 42 days reduced ventral prostatic epithelial height. TE increased ventral prostatic epithelial height and duct luminal area. These increases by TE were reduced after treatment with Red Maca for 42 days. Histology pictures in rats treated with Red Maca plus TE were similar to controls. Phytochemical screening showed that aqueous extract of Red Maca has alkaloids, steroids, tannins, saponins, and cardiotonic glycosides. The IR spectra of the three ecotypes of Maca in 3800-650 cm (-1) region had 7 peaks representing 7 functional chemical groups. Highest peak values were observed for Red Maca, intermediate values for Yellow Maca and low values for Black Maca. These functional groups correspond among others to benzyl glucosinolate.

**Conclusions:**

Red Maca, a cruciferous plant from the highland of Peru, reduced ventral prostate size in normal and TE treated rats.

## Background

*Lepidium meyenii*, a traditional Peruvian cruciferous vegetable known as Maca, grows exclusively at altitudes over 4000 m. The hypocotyl, the edible part of the plant, is used as a nutritional supplement and for its enhancing sperm production properties [1]. Maca is presented in different ecotypes according to the colors of its hypocotyls, ranging from white to black [2].

Epidemiological studies have found that consumption of cruciferous vegetables is associated with a reduced risk of prostate cancer [3-5]. Cruciferous (Brassica) vegetables are broccoli, cabbage, mustard and collard greens, bok choy [3], and members of the genus *Lepidium *[1,6], including Maca.

In the many hundreds of cruciferous species investigated, all are able to synthesize glucosinolates [7]. Glucosinolate content in cruciferous vegetables is highly variable, depending of plant age and environmental factors which can cause the broad range of values reported to vegetables of the same variety [8]. This variability may explain differences in epidemiological studies related to protection of intake of cruciferous vegetables against prostate cancer [3-5,9-11].

Upon ingestion by humans [12] or rats [13], glucosinolates are converted in isothiocyanates by gut microflora. As alternate mechanism, glucosinolates are catalyzed by dietary myrosinase [13].

Almost all of the mammalian chemoprotective activity from cruciferous is due to these isothiocyanates [7,14]. Therefore, glucosinolates after conversion to isothiocyanates have both antiproliferative and proapoptotic properties in prostate cells [14-18]. The isothiocyanates formed from aromatic glucosinolates decompose spontaneously to indole-3-carbinol (I3C) [7]. I3C and metabolites induce apoptosis in human prostate cancer cells [16-18].

The absolute content of glucosinolates in fresh Maca hypocotyls is relatively higher than reported in other cruciferous crops [19]. The most abundant glucosinolates detected in Maca were the aromatic glucosinolates: benzyl glucosinolate (glucotropaeolin) [19-21]; as a result, it is possible that *Lepidium meyenii *(Maca) may have important effects on prostate. More recently, it has been demonstrated that an integral suspension of *Lepidium latifolium *significantly reduced prostate size and volume in castrated rats where the hyperplasia was induced by steroid treatment [6]. For such reason, the present study has been designed to determine the effect of three ecotypes of Maca on ventral prostate of rats.

## Methods

### Animals

Adult Holtzman rats were obtained from our Animal House at the Universidad Peruana Cayetano Heredia and used for the present study. The rats were maintained 4–6 per cage at environmental temperature (22°C) with a 12:12 h light/dark cycle. Also they were fed with Purina laboratory chow and tap water *ad libitum*. All animal experiments were conducted in compliance with "Guide for the care and use of laboratory animals" of the National Institutes of Health from the USA [22]. The Institutional Review Board of the Scientific Research Office from the Universidad Peruana Cayetano Heredia approved the study.

### Experimental protocol

#### Experiment 1: Effect of different ecotypes of Maca on prostate size in rats

Rats were treated with vehicle, Yellow Maca, Red Maca or Black Maca for 7 days in dose of 2 g dried Maca hypocotyls/Kg BW. This dose was selected from a previous dose response study [23]. Each Maca treated group included 12 animals, and Control (vehicle) sample size included 35 animals. Maca or vehicle was orally administered using an intubation needle No 18 (Fisher Scientific, Pittsburgh, Pennsylvania).

#### Experiment 2: Effect of Red Maca on prostate size and histology in rats treated with testosterone enanthate (TE)

Rats were injected (i.m) with 0.1 ml (25 mg) of testosterone enanthate (TE) on day 1 and day 7. Control rats received 0.1 ml oil (im) at day 1 and at day 7. A group treated with TE received also Red Maca (2 g/Kg) for 14 days and another group treated with TE received Red Maca for 42 days. Control rats received vehicle by oral route for 14 or 42 days. Oral treatment (Maca or vehicle) and intramuscular treatment (TE or vehicle) both started on day 1.

### Preparation of aqueous extract of Lepidium meyenii (Maca)

The dried hypocotyls of *Lepidium meyenii *were obtained from Carhuamayo, Junin at 4000 m altitude. The ages of different Maca plants were similar. All hypocotyls were obtained at the same time. Irma Fernandez, a Botanist of the Department of Pharmaceutical Sciences at Universidad Peruana Cayetano Heredia, authenticated the identity of the plant by visual inspection. The biological activity of the plant is located in the hypocotyls, which are consumed by natives after natural drying. Traditionally, the dried hypocotyls of Maca are boiled and served as juice.

For the present study, the aqueous extract of the hypocotyls was prepared according to the traditional method. First, 500 g of the dried hypocotyls were pulverized and placed in a container with 1500 ml of water, and boiled for 120 minutes. Next, the preparation was left standing to cool, and then it was filtered. Finally, the filtrate containing 333 mg of dry Maca hypocotyls in 1 ml was placed in small vials and kept in 4°C refrigerator.

### Sacrifice, Blood and sample of tissue

One day after the last treatment, rats were sacrificed by decapitation. Blood sample was obtained from cervical trunk from rats treated for 7 days with different ecotypes of Maca. Blood samples were centrifuged at 1,000 g, and sera were separated, placed in vials and kept frozen until assayed for sex hormone levels. Ventral prostate was used for histological study.

### Organ Weights

After animals were sacrificed several organs (testes, epididymis, ventral prostate, seminal vesicles, kidneys, liver, spleen, heart and lungs) were collected, dissected free of fat, and weighed.

### Measurement of serum estradiol and testosterone

Serum estradiol and testosterone concentrations were measured by radioimmunoassay using commercial kits (Diagnostic Products Co, Los Angeles, USA). The hormone labeled with iodine-125 was used as radioactive marker. Samples were run in the same assay to avoid inter-assay variation. The intra-assay variation was 6.42% for estradiol, and 5.5% for testosterone. Sensitivity of testosterone assay was 4 ng/dl and for estradiol assay was 8 pg/ml.

### Histological study

Ventral prostate lobes obtained from experiment 2 were excised and dissected free of fat. Ventral prostates (VP) were immersion-fixed in Bouin's fixative. After their dehydration, VP were embedded in paraffin. The tissue blocks were sectioned into 5 um thickness and stained with hematoxylin and eosine (H&E), and then observed under a light microscope.

Epithelial height (um) and duct lumen area (um^2^) were measured by sampling 10 random sections per slide in the peripheral region of the ventral prostate. In each duct, 20 cells were measured for epithelial height. All assessments were performed using an axiostar plus microscope (Carl Zeizz, Thornwood, New York, USA). The images were captured by a Moticam2000 (Richmond, B.C, Canada) coupled to a personal computer. Motic image plus 2.0 software (Motic Instruments Inc.) was used for measurements of prostatic epithelial height and duct luminal area and calculated by statistic ANOVA test. Pictures at 50× and 400× magnifications are included.

### Phytochemistry of Red Maca

The phytochemical screening in the aqueous extract of Red Maca was performed using standard phytochemical procedures [24,25]. Maca aqueous extract was lyophilized previously to extraction procedures. After extraction in methanol or ethanol, the presence of alkaloids (Dragendorff reagent; Mayer's test), flavonoids (Shinoda test), steroids (Liebermann-Burchard/Thin Layer Chromatography test), anthraquinones (Bornträger reaction), tannins (Gelatin/Ferric chloride test), saponins (Froth test), sesquiterpene lactones (Vainillin test and Ferric hydroxamate test), coumarins (Vainillin test and Ferric hydroxamate test), cardiotonic glycosides (Raymond reagent), and cardenolids (Kedde reagent) were assessed [24,25].

### Measurement of infrared (IR) spectra

IR spectra of lyophilized aqueous extracts of Red, Yellow and Black Maca were measured from 3800 cm^-1 ^to 650 cm^-1 ^with an FT-IR spectrophotometer (SPECTRUM2000, Perkin Elmer Ltd., Beaconsfield, England). An overhead-attenuated total refraction (ATR) accessory was equipped as the sample stage for solid samples. All spectral measurements were done at 1 cm^-1 ^resolutions. Data are presented as absorbance units. Each peak represents the presence of a functional chemical group. Differences in the height of absorbance peaks reflect differences in amount of functional groups.

### Statistical analysis

Data were analyzed using the statistical package STATA (version 8.0) for personal computer (Stata Corporation, 702 University Drive East, College Station, TX, USA).

Data are presented as mean ± standard error of the mean (SEM). Homogeneity of variances was assessed by the Bartlett test. If variances were homogeneous, differences between groups were assessed by analysis of variance (ANOVA). If F value in the ANOVA test was significant, the differences between pair of means were assessed by the Scheffeé test. If variances were non homogeneous, non parametric tests were used.

A value of *P *< 0.05 was considered statistically significant.

## Results

### Red Maca but not Black or Yellow Maca reduced ventral prostate weight in rats

**Figure 1:**
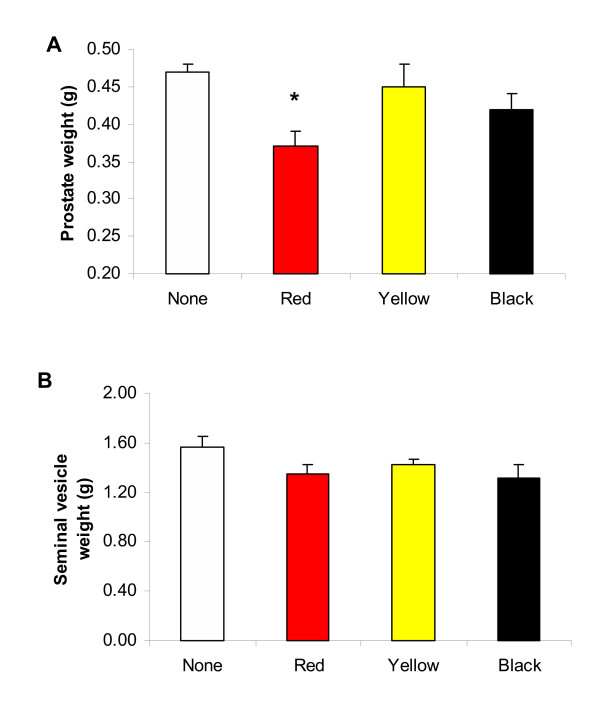
Ventral prostate (A) and seminal vesicles (B) weights in adult rats treated for 7 days with different ecotypes of Maca (2 g/kg BW). Data are Mean ± SEM *P < 0.05 with respect to control value. Number of animals was 35 for controls, 12 for Yellow Maca, 12 for Red Maca and 12 for Black Maca. None: control group receiving vehicle.

Ventral prostate weight was significantly reduced in rats treated for 7 days with Red Maca (P < 0.05). Black Maca and Yellow Maca did not modify ventral prostate weight (Figure 1a). Seminal vesicles weights were not modified by treatment with any ecotype of Maca (Figure 1b). Body weight was similar in the control group (415.8 ± 3.3 g, mean ± SEM) and in rats treated with Red (407.5 ± 7.1 g), Yellow (421.5 ± 6.1 g) or Black Maca (426.5 ± 6.8 g).

### Maca and serum sexual hormone levels

**Figure 2:**
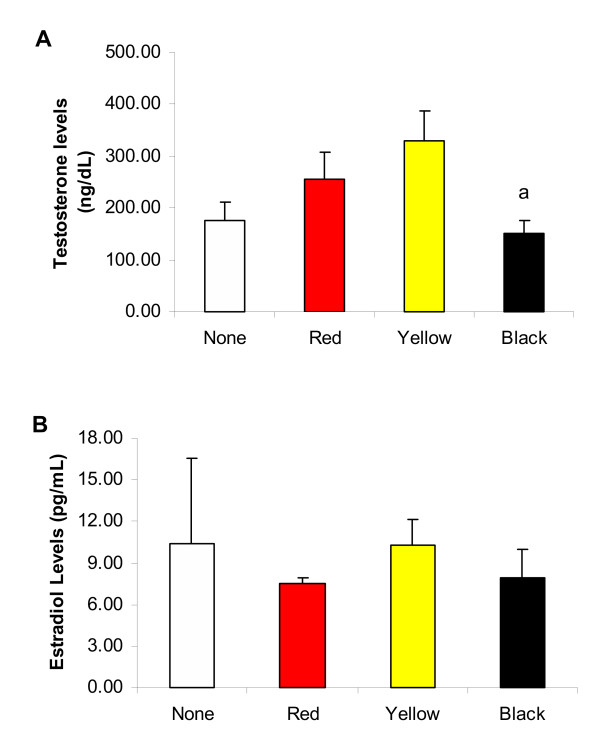
Effects of different Maca ecotypes administered for 7 days on serum testosterone (A) or estradiol (B) levels in rats. Data are mean ± SEM. Maca (2 gr/Kg BW) was administered for 7 days. Number of rats was 10 in the control group, 6 in the Red Maca treated group, 6 in the Yellow Maca, and 6 in the Black Maca treated group. P:NS between groups treated with Maca and control rats. ^a^P < 0.05 with respect to the Yellow Maca treated group.

Means of serum testosterone and estradiol levels were similar between control group and groups treated for 7 days (Figure 2a,b) with Red, Yellow or Black Maca. The group treated with Yellow Maca showed higher serum testosterone levels than the group treated with Black Maca (P < 0.05). Higher serum testosterone levels in the group treated with Yellow Maca were due to two rats with high serum testosterone levels.

### Red Maca reduced ventral prostate weight in rats treated with testosterone enanthate (TE)

**Figure 3:**
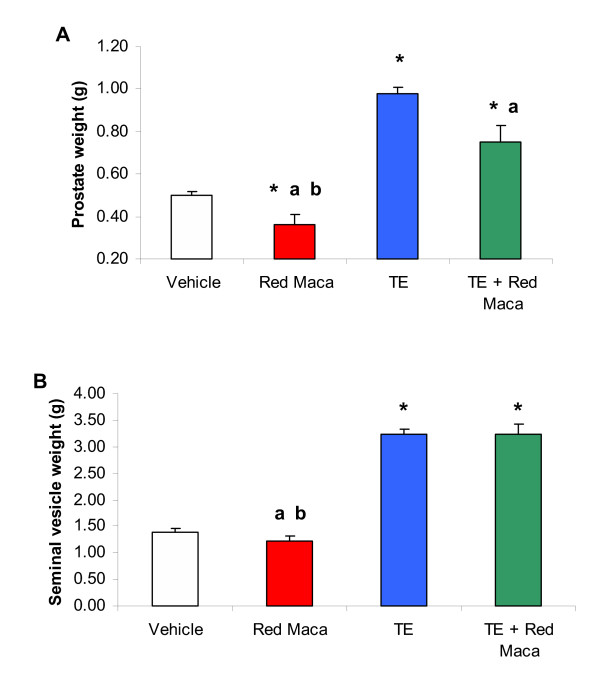
Ventral prostate (A) and seminal vesicles (B) weights in adult rats treated for 14 days with Red Maca. Data are mean ± SEM.TE: rats treated on day 1 and 7 with testosterone enanthate (25 mg each) i.m, Red Maca (2 g/Kg BW) was given orally during 14 days. Rats were sacrificed on day 15. * P < 0.05 with respect to vehicle group; ^a^P < 0.05 with respect to TE group. ^b^P < 0.05 with respect to the group treated with TE+Red Maca. Number of rats was 13 for the control group, 6 for the Red Maca, 6 for TE, and 6 for the TE plus Red Maca groups.

**Figure 4:**
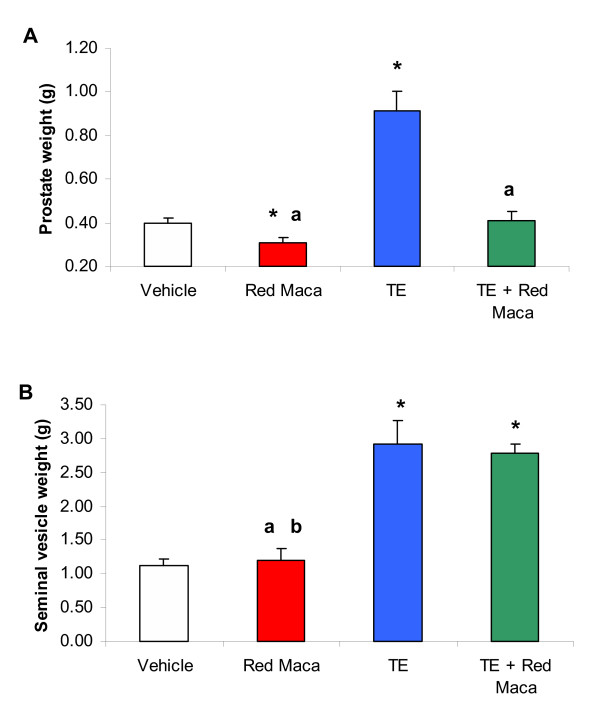
Ventral prostate (A) and seminal vesicles (B) weights in adult rats treated for 42 days with Red Maca. Data are mean ± SEM.TE: rats treated on day 1 and 7 with testosterone enanthate (25 mg each) i.m. Red Maca (2 g/Kg BW) was given orally during 42 days. Rats were sacrificed on day 43. *P < 0.05 with respect to vehicle group; ^a^P < 0.05 with respect to TE group. ^b^P < 0.05 with respect to the group treated with TE+Red Maca. Number of animals was 12 in the control group, 7 in the Red Maca group, 5 in the TE group and 5 in the TE plus Red Maca group.

Treatment with TE increased significantly ventral prostate weight almost to double value of the control group (P < 0.05) (Figures 3a and 4a). The increase in ventral prostate weight was maintained in high levels up to 5 weeks after last TE injection. Seminal vesicles weight increased 2.5 times that control values (P < 0.05) (Figures 3b and 4b). Treatment with only Red Maca for 14 days (P < 0.05) (Figure 3a) or 42 days (P < 0.05) (Figure 4a) resulted in low ventral prostate weight compared with control values. Seminal vesicles values were not affected after treatment with Red Maca for 14 or 42 days (Figures 3b and 4b).

Red Maca administered for 14 days also reduced ventral prostate weight in rats treated with TE (P < 0.05). The effect was more noticeable after 42 days of treatment with Red Maca (P < 0.05). After 42 days of treatment with Red Maca, the ventral prostate weight was reduced more than 50% (Figure 4a). After 14 or 42 days of treatment with Red Maca, the increased seminal vesicles weights induced by TE was not affected (Figures 3b and 4b).

Ventral prostate weight related to body weight was lower in the group treated for 14 days with Red Maca (0.11 ± 0.01 g/100 g BW) than in controls (0.15 ± 0.006 g/100 g BW) (P < 0.05). Rats treated with TE plus Red Maca had lower prostate weight related to BW (0.23 ± 0.02 g/100 g BW, P < 0.05) than rats treated with TE (0.29 ± 0.01) but prostate weight relative to BW was still higher in rats treated with TE plus Red Maca than in controls (P < 0.05). The same pattern was observed when rats were treated for 42 days with Red Maca (Data not shown).

Rats treated with two TE injections showed lower body weight at day 42 after the first injection (P < 0.05) compared to controls (290.63 ± 30.48 g and 383.60 ± 11.31 g in TE and vehicle treated groups). This low body weight was also observed in the group treated with TE plus Red Maca (319.30 ± 3.08 g) (P < 0.05).

Weights of testes, epididymis, kidneys, liver, spleen, lungs and heart were not affected by treatment with Red Maca for 7, 14 or 42 days (Data not shown).

### Histological study

**Figure 5:**
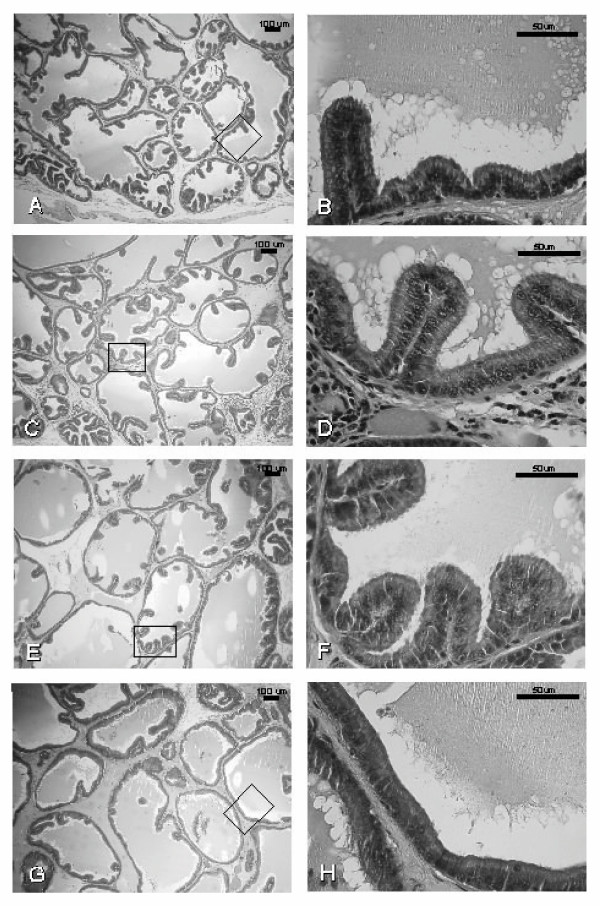
The effects of Red Maca administered to rats for 14 days on ventral prostatic epithelial height and duct luminal area. **A,B**: Control group; **C,D**: Red Maca treated; **E,F**: TE treated; **G,H**: TE+Red Maca treated. HE stain. Left: ×50 magnification; Right: ×400 magnification.

**Figure 6:**
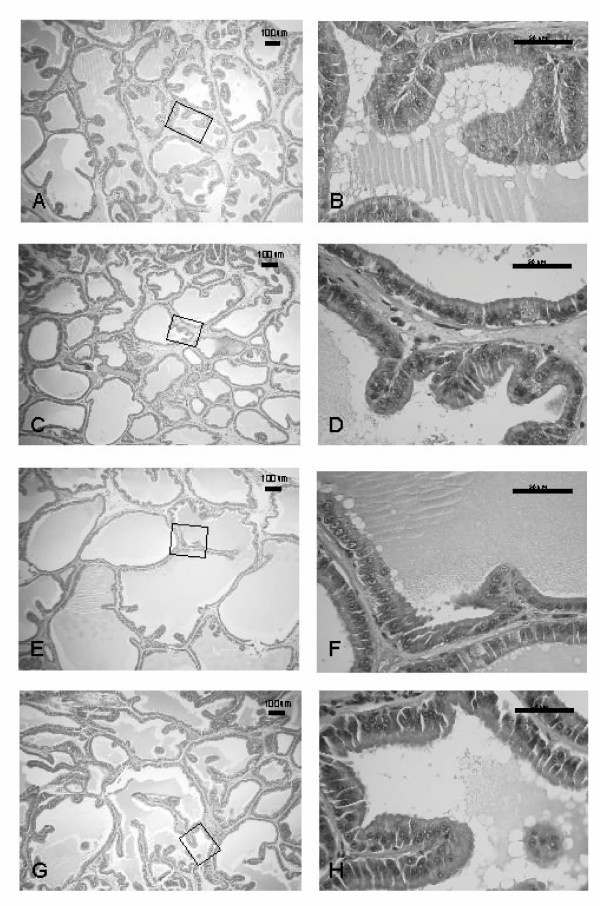
The effects of Red Maca administered to rats for 42 days on ventral prostatic epithelial height and duct luminal area. **A,B**: Control group; **C,D**: Red Maca treated; **E,F**: TE treated; **G,H**: TE+Red Maca treated. HE stain. Left: ×50 magnification; Right: ×400 magnification.

Sections of ventral prostate in the peripheral region of the ductal system after 14 and 42 days of treatment with vehicle (control), Red Maca, ET, or ET plus Red Maca are shown in Figures 5 and 6.

At 50× magnification, the number of ducts per field from rats treated with Red Maca for 14 days was slightly higher than in controls (Figures 5a and 5c). TE reduced the number of ducts per field as a consequence of increase in duct area (Figure 5e). Red Maca increased the number of ducts per field in rats treated with TE (Figure 5g). At 400× magnification, in Red Maca treated rats (alone or with TE), cell size was decreased and membrane blebbing and nuclear distortion are apparent (Figures 5d and 5h vs Figures 5b and 5F).

At 50× magnification, compared to control (Figure 6a), the treatment with Red Maca for 42 days resulted in high number of ducts per field by Maca effect reducing the lumen area (Figure 6c). Treatment with TE resulted in lower number of ducts per field as a result of testosterone induced high luminal area (Figure 6e). Treatment with TE plus Red Maca (Figure 6g) compared to TE (Figure 6e) showed an increase in the number of ducts per field. At higher magnification (400×), it was observed secretory luminal cells lined with a single layer of columnar epithelium in the control group (Figure 6b). In specimens treated with only Red Maca, the epithelium of the ventral prostate showed a change from columnar to cuboidal shape (Figure 6d). TE caused an increase in proliferation of epithelial cells (Figure 6f). Red Maca reduced the epithelium in rats treated with TE (Figure 6h). Membrane blebbing and nuclear distortion are apparent in rats treated with Red Maca (Figures 6d and 6h).

**Figure 7:**
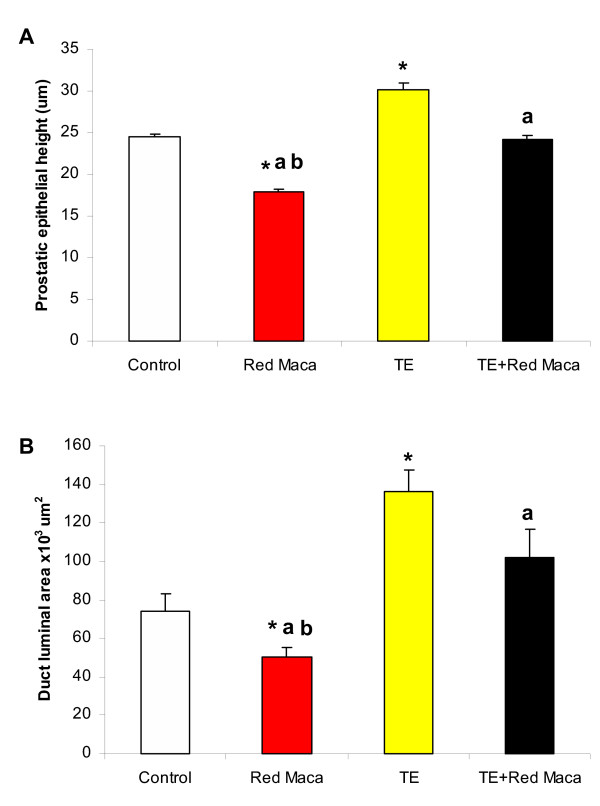
Ventral prostatic epithelial height (A) and luminal area (B) in control rats, rats treated with Red Maca (RM) alone, testosterone enanthate (TE) alone or TE+Red Maca. Rats were treated for 42 days. *P < 0.05 with respect to control, ^a^P < 0.05 respect to TE group; ^b^P < 0.05 respect to TE+ Red Maca. Differences in duct luminal areas were assessed with Mann-Whitney U test.

Quantitative analyses of epithelial height and luminal area in rats treated for 42 days are presented in Figure 7. Rats treated for 42 days with only Red Maca showed lower prostatic epithelial height (P < 0.05) and duct luminal area (P < 0.05) than control, TE and TE+Red Maca groups (Figure 7a–b).

Rats treated during 2 weeks with injections of TE once a week showed higher prostatic epithelial height (P < 0.05) and duct luminal area (P < 0.05) at day 42 compared to controls (Figure 7a–b). Rats treated with TE plus Red Maca for 42 days showed that prostatic epithelial height and luminal area were similar to those observed in the control group (Figures 7a–b).

### Phytochemistry of Red Maca

The phytochemical analysis of the ethanolic extract prepared from lyophilized aqueous extract of Red Maca hypocotyls revealed the presence of alkaloids, steroids, saponins and cardiotonic glycosides, and the absence of flavonoids, anthraquinones, tannins, sesquiterpene lactones and coumarins (Table 1).

**Table 1:** Result of phytochemical screening of extracts of Red Maca.

**Tests**	**Ethanolic extract**	**Methanolic extract**
**Alkaloids**		
Dragendorff test	+	+
Mayer's test	+	+
**Flavonoids**		
Shinoda test	-	-
**Steroids**		
Liebermann-Burchard test	+	+
**Anthraquinones**		
Bornträger test	-	-
**Tannins**		
Gelatin/Ferric Chloride test	-	+
**Saponins**		
Froth test	+	+
**Sesquiterpene Lactones**		
Ferric hydroxamate test	-	-
Vainillin test	-	-
**Coumarins**		
Ferric hydroxamate test	-	-
Vainillin test	-	-
**Cardiotonic glycosides**		
Raymond test	+	NA
**Cardenolids**		
Kedde test	NA	-

-, test negative; +, test positive; NA, not available

The phytochemical analysis of the methanolic extract prepared from lyophilized aqueous extract of Red Maca hypocotyls revealed the presence of alkaloids, steroids, tannins and saponins, and the absence of flavonoids, coumarins, anthraquinones, sesquiterpene lactones and cardenolides (Table 1). The positive tests were more intense for alkaloids than for the other compounds.

**Figure 8:**
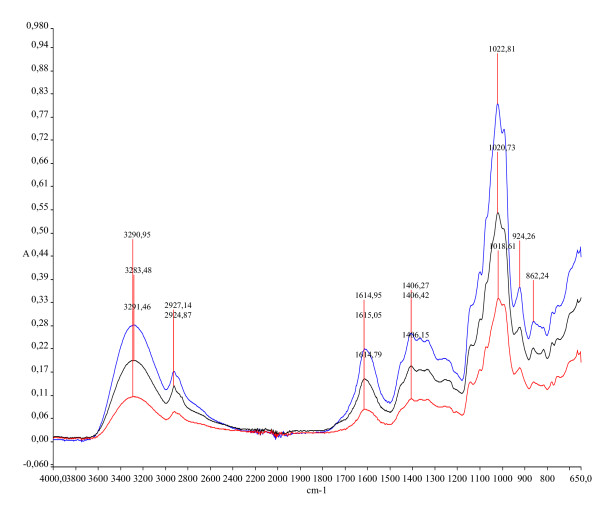
Infrared (IR) spectra of lyophilized aqueous extract of three ecotypes of *Lepidium meyenii *(Maca). Data are expressed in absorbance units (A). Wave number is expressed in cm^-1^. IR spectra were measured from 4000 cm^-1 ^to 650 cm^-1 ^with a FT-IR spectrophotometer equipped with an ATR apparatus. Highest absorbance values correspond to Red Maca, intermediate values to Yellow Maca and lowest values to Black Maca. Peaks of absorbance are recorded at 3291 cm^-1^, 2927 cm^-1^, 1614 cm^-1^, 1406 cm^-1^, 1022 cm^-1^, 924 cm^-1 ^and 862 cm^-1^.

**Figure 9:**
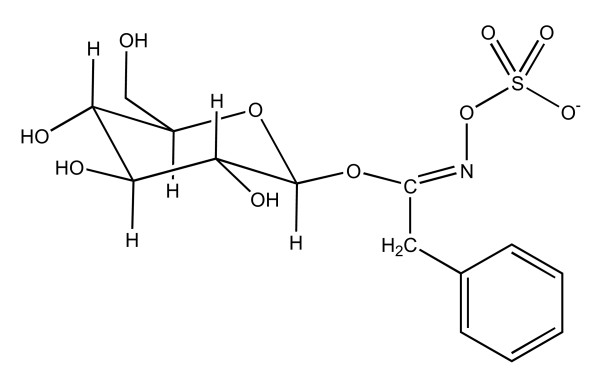
Structure of Glucotropaeolin (Benzyl glucosinolate)

The IR spectra of the three Maca's ecotypes extracts are shown in Fig. 8. The IR spectra of the three ecotypes of Maca in 3800-650 cm^-1 ^region had 7 peaks, which were at 3291 cm^-1^, 2927 cm^-1^, 1614 cm^-1^, 1406 cm^-1^, 1022 cm^-1^, 924 cm^-1 ^and 862 cm^-1^. These peaks are due to C-H, OH, amides, amines, carboxylic acids, aromatic, and alkyls groups, respectively. Highest peak values were observed for Red Maca, intermediate values for Yellow Maca and low values for Black Maca. These functional groups correspond among others to benzyl glucosinolate (Figure 9).

## Discussion

The present study was designed to determine if different ecotypes of *Lepidium meyenii *(Maca), a cruciferous plant that grows exclusively over 4000 m in Peruvian Andes, affect ventral prostate size. It was of great interest to demonstrate that Red Maca reduced significantly ventral prostate weight. This effect was not observed after treatment with Yellow or Black Maca. The effect of Red Maca was specific for prostate, since other organs as testes, epididymis, seminal vesicles, kidneys, spleen, liver, lungs and heart were not affected.

It was also demonstrated an effect of Red Maca on rats in which ventral prostate size was enlarged by two injections of testosterone enanthate. In fact, Red Maca administered for 14 or 42 days reduced the effect of TE. At 42 days, the ventral prostate size of rats treated with TE plus Red Maca was similar to that of control rats treated only with vehicle. Epithelial height and luminal areas were proved to be sensitive parameters for the evaluation of androgen effects on prostates [26]. The present study shows that prostatic epithelial height increased after treatment with TE. The same effect has been observed when castrated rats were treated with testosterone [26] suggesting that prostatic epithelial height is androgen dependent. Red Maca was able to reduce the prostatic epithelial height of TE treated rats. This would means that Red Maca interferes the androgen action.

Growth of the prostate is a hormone-mediated phenomenon regulated by both androgens and estrogens [27]. However, data showed that Red Maca affect ventral prostate size without affecting serum testosterone or estradiol levels. This is not surprising because previously, it has been published that dietary phytoestrogens may affect prostate size without modify circulating testosterone or estrogen level [28], but affecting the androgen action in the rat prostate [27]. Our data on effect of Red Maca on ventral prostate size in rats previously treated with testosterone enanthate suggest that this cruciferous is acting by interfering the androgen action. Maca is characterized by its higher content on aromatic glucosinolates [19-21]. Recently, it has been described a metabolite of the aromatic glucosinolates that specifically antagonizes androgen receptor [18]; therefore, it is possible that effect of Red Maca on ventral prostate size may be due in part to an action of glucosinolate metabolites on androgen receptor. However, further studies will be required to clarify mechanism of action of this cruciferous plant.

Recently, increasing evidence has been presented suggesting that cruciferous (Brassicas) vegetables may reduce the risk of prostate cancer development [3,4]. The genus *Lepidium *could be an important alternative for treatment of prostate diseases. Other Brassica from the genus *Lepidium*, as *Lepidium latifolium *reduced prostate weight [6] suggesting that cruciferous from the genus *Lepidium *may have important anti-proliferative and proapoptotic effects. In Red Maca treated rats, cell size has decreased and membrane blebbing and nuclear distortion are apparent suggesting a pro-apoptotic effect.

It is still unknown the active principle for the effect of Red Maca on ventral prostate and why the action is specific since any other organ was affected. Moreover, the different effects among ecotypes seem to be due to different amount of active metabolites.

This study used aqueous extract of dried hypocotyls of *Lepidium meyenii*. In the aqueous extract is possible to find glucosinolates [7] and anthocyanines [29]. Both compounds have antiproliferative and proapoptotic properties in prostate cancer cells [14-18,30]. As effect was specific for Red Maca and not for Yellow or Black Maca, it is probably that Red Maca has more glucosinolate content than other ecotypes. Results from the infrared (IR) spectroscopy showed that peaks of absorbance were higher for Red Maca, intermediate for Yellow Maca and lower for Black Maca. Each peak reflects specific chemical functional groups. Several functional groups found in the different Maca ecotypes correspond among others to benzyl glucosinolate. In such sense, it is suggested that benzyl glucosinolate content is higher in Red Maca, intermediate in Yellow Maca and lower in Black Maca. In addition to the potential glucosinolates effects on prostate, it is possible that other active metabolites may be acting on prostate. Maca aqueous extracts were further extracted with ethanol or methanol and assessed for different compounds. The compounds found are potential candidates to affect prostate; however, it is difficult at this time to ascertain which specific compound has the prostate effect. In fact, the phyto-chemical screening data showed that aqueous extract of Red Maca has alkaloids, steroids, tannins, saponins and cardiotonic glycosides, all of them may have effects on prostate. Phytochemical study showed that Red Maca was more positive for alkaloids than from other compounds. The alkaloid, (1R,3S)-1-methyltetrahydro-β-carboline-3-carboxylic acid has been reported as a constituent of Maca [20]. This alkaloid acts as antioxidants and free radical scavengers [31]. Beta carbolines are also proapoptotic compounds [32] and they have antitumor activities [33]. Further studies will be required to determine the impact of tetrahydro-beta-carbolines from Maca on prostate.

## Conclusions

Indeed, the data presented here show that Red Maca reduced ventral prostate size in normal adult rats and also in rats treated with testosterone enanthate. Hence, it is proposed that Red Maca may have important implications under pathological conditions of the prostate.

## Authors' contributions

GFG conceived of the study participating in its design, coordination, and drafting the manuscript.

SM participated in the design of the study and the study of different ecotypes of Maca.

JN participated in the biological study with different ecotypes of Maca.

GF participated in the phytochemical screening

JR participated in the statistical analysis

SY participated in the histological study

PY participated in the histological study

MG participated in the design and analysis of results, and its interpretation.

All authors read and approved the final manuscript.
